# Radial artery intima-media thickness regresses after secondary prevention interventions in patients’ post-acute coronary syndrome and is associated with cardiac and kidney biomarkers

**DOI:** 10.18632/oncotarget.18511

**Published:** 2017-06-16

**Authors:** Damilola D. Adingupu, Helena U. Westergren, Santosh Dahgam, Ann-Cathrine Jönsson-Rylander, Juuso Blomster, Per Albertsson, Elmir Omerovic, Sara Svedlund, Li-Ming Gan

**Affiliations:** ^1^ AstraZeneca R&D Gothenburg, Mölndal, Sweden; ^2^ Department of Molecular and Clinical Medicine, Institute of Medicine, Sahlgrenska Academy at the University of Gothenburg, Gothenburg, Sweden; ^3^ Department of Clinical Physiology, Sahlgrenska University Hospital, Gothenburg, Sweden; ^4^ Department of Cardiology, Sahlgrenska University Hospital, Gothenburg, Sweden

**Keywords:** atherosclerosis, cardiovascular, intima-media thickness, inflammation, ultrasound

## Abstract

**Background:**

Radial artery intima-media thickness (rIMT) measured by ultra-high-resolution ultrasound is associated with increased cardiovascular risk and predicts outcomes. We performed non-invasive high-resolution ultrasound of the radial artery to investigate vascular changes in subjects presenting with acute coronary syndrome (ACS) and who had undergone percutaneous coronary intervention (PCI).

**Purpose:**

In the present work, we aimed to follow rIMT change over time post-acute coronary syndrome as a tool to monitor potential response to intensified medical therapy.

**Methods:**

We examined 256 subjects who underwent PCI due to ACS and healthy controls (n= 39) and we measured a number of biomarkers, which are known to be associated with cardiovascular disease. Images of radial artery were acquired bilaterally in the longitudinal view using a 50 MHz transducer (Vevo 2100 VisualSonics, Inc, Toronto, Ontario, Canada). Carotid IMT (cIMT) and rIMT were measured at <1 month after index PCI followed by a repeated measurement of rIMT at 4 months from the ACS in a sub-set (n=117).

**Results:**

rIMT measured within 1 month post ACS was significantly higher than rIMT after 4 months from ACS, (p < 0.0001), mean ± SD (rIMT right 0.35 ± 0.08; rIMT left 0.37 ± 0.08) vs. (rIMT right 0.29 ± 0.08; rIMT left 0.31 ± 0.09) respectively. There was no statistically significant change in cIMT. In healthy controls there were no changes in rIMT or cIMT overtime. High levels of CX3CL1 and myeloperoxidase measured within one month post ACS are associated with increase of rIMT, r=0.38 (p< 0.0001) and r=0.41 (p< 0.0001) respectively.

**Conclusions:**

rIMT seem to decrease systemically after ACS and is accompanied with corresponding biomarker change. The cause and clinical implications of the observed decrement in rIMT after ACS need further studies.

## INTRODUCTION

Atherosclerosis is a systemic disease which alters the arteries of different vascular beds and is characterized by inflammation [1] and thickening of the intimal and medial layers of the arterial wall [2]. There are various imaging techniques used in the investigation of atherosclerosis in the arterial wall with an emphasis in coronary imaging to assess coronary artery disease as the most significant manifestation of atherosclerotic diseases. Revascularization of coronary artery stenosis leads to both improved quality of life and prolonged survival [3]. Percutaneous coronary intervention (PCI) is however mostly based on morphological assessment of coronary angiography, without direct measure of potential coronary flow limiting properties of these vascular changes. Besides clinical outcome, it is important to be able to assess early the individual response to treatment, which may enable us to identify patients who require further intervention or more intensive medical therapy, thus improving long-term results.

It has been shown that atherosclerosis perturbs the radial arteries [4, 5], which carry similar characteristics to coronary arteries. On the other hand carotid intima media thickness (cIMT) is a well validated and accepted marker of atherosclerosis [6, 7], however it has poor resolution [8] in comparison to radial artery intima-media thickness (rIMT). Furthermore, the sensitivity of cIMT to predict total carotid plaque area has been questioned [9]. We have shown previously that non-invasively measured rIMT using ultra-high-resolution ultrasound is a surrogate marker for atherosclerosis, is associated to cIMT and correlates with various cardiovascular risk factors [10]. Furthermore, rIMT is associated with age, increased cardiovascular risk and predicts outcomes [10, 11] in patients with suspected coronary artery disease. Radial intima thickening has been shown to be present early in the development of hypertension, which is an important risk factor for cardiovascular disease [12]. The radial artery can be studied with relatively less discomfort to patients compared with cIMT, and used on participants as young as 10 years of age [13].

There is a need for imaging technique that is able to detect vascular changes in relatively small patient cohorts, not time-consuming, highly reproducible and can provide specific result in a relatively short time-scale, and thereby can be used in future intervention trials as a response-to-treatment marker. With this respect we investigated temporal changes in rIMT and inflammation biomarkers directly after acute coronary syndrome (ACS) and with repeated measurements in stabilized phase, 4 months after ACS. Kidney impairment and chronic inflammation are known to add incremental risk in individuals with heart failure and ischemic diseases [14, 15], therefore we examined the relation between rIMT, creatinine, cystatin C and inflammatory biomarkers E-selectin, CX3CL1 and MPO.

## RESULTS

### Patient characteristics

A total of 256 subjects with mean age 65 ± 9 years, 17% women and 83% men were included in the study, and 117 subjects underwent repeated rIMT at 4 months post ACS during the time period of 4 years. The median time to visit 1 from the day PCI was performed was 13 days, and the median time to visit 2 was 19 weeks. Demographic data and medication use are shown in Table 1. Briefly, there were no significant differences in age, clinical characteristics, medication use nor clinical investigations between the whole study cohort and the sub-set having complete measurement of rIMT at visit 1 (<1 month post ACS), Table 1. There was a significant difference in age between healthy controls, whole study cohort and the sub-set cohort (p= 0.0282), which was attributable to healthy controls been younger (Table 1). Biochemical and lipid profile data are presented in Table 2. There were no detectable differences in the sub-set analysis. Lipid profile was improved at visit 2 in ACS subjects, and unchanged in healthy controls.

**Table 1:** Clinical characteristics and medication use of subjects within 1 month of presenting with ACS and healthy controls at baseline visit

Parameter	Cohort (n = 256)	Sub-set (n = 117)	Healthy controls (n=39)
Age (years)	64.5 ± 8.6	64.5 ± 8.9	60 ± 3.5
Men (%)	82.3	85.3	76.9
BMI (kg/m2)	25.8 ± 6.8	25.8 ± 4.7	24.8 ± 2.7
Smokers (% Never, Ex, Current)	42.7, 35.9, 21.4 (n=206)	41.1, 38.3, 20.6 (n= 107)	66.7, 28.2, 5.1
Snuffing (% Never, Ex, Current)	88.4, 6.3, 5.3 (n=189)	87.9, 8.1, 4.0 (n= 99)	-
Diabetes (% Yes)	9.7 (n=217)	7.1 (n= 114)	-
Hypertension (% Yes)	45.2 (n= 217)	48.2 (n= 114)	-
Hyperlipidemic (% Yes)	28.6 (213)	28.6 (n= 112)	-
Previous CABG (% Yes)	3.8 (n= 216)	2.6 (n= 114)	-
Previous PCI (% Yes)	13.0 (n= 216)	13.8 (n= 114)	-
**Medication use at the time of ACS**			
Aspirin (% Yes)	22.9 (n= 214)	25.0 (n= 113)	-
Beta-blockers (% Yes)	21.7 (n= 212)	22.4 (n= 111)	-
ACE inhibitors (% Yes)	11.7 (n= 213)	12.9 (n= 112)	-
Angiotensin II blocker (% Yes)	12.7 (n= 213)	11.2 (n= 112)	-
Statins (% Yes)	21.1 (n= 213)	21.6 (n= 113)	-

Data are expressed as mean ± SD for continuous variables and as percentages for categorical variables. ACS: acute coronary syndrome; BMI: body mass index; CABG: coronary artery bypass grafting; PCI: percutaneous coronary intervention. Healthy controls had no previous history of cardiovascular disease and were on no medication at the time of enrolment into the study.

**Table 2:** Hemodynamic and lipid profile measures taken within 1 month of ACS and at baseline for healthy controls (visit 1) and at visit 2

		Total cholesterol (mg/dl)	LDL-cholesterol (mg/dl)	HDL-cholesterol (mg/dl)	Triglycerides (mg/dl)	Systolic BP(mm Hg)	Diastolic BP(mm Hg)
Visit 1	Cohort (n = 256)	3.85 ± 0.92	2.26 ± 0.76	1.17 ± 0.31	1.19 ± 0.75	126 ± 26	74 ± 16
	Sub-set (n = 117)	3.85 ± 0.79	2.27 ± 0.67	1.15 ± 0.35	1.09 ± 0.57	125 ± 25	74 ± 15
	Healthy control (n= 39)	5.28 ± 0.62	3.54 ± 0.55	1.51 ± 0.38	1.07 ± 0.39	131 ± 17	81 ± 10
Visit 2	Cohort (n = 249)	3.76 ± 0.85*	2.09 ± 0.71***	1.29 ± 0.33***	1.21 ± 0.94	132 ± 21*	77 ± 12*
	Sub-set (n = 113)	3.86 ± 0.88	2.17 ± 0.71**	1.30 ± 0.32***	1.16 ± 0.86	130 ± 20	75 ± 13
	Healthy control (n= 37)	5.33 ± 0.64	3.60 ± 0.58	1.56 ± 0.36	0.96 ± 0.38	124 ± 15**	80 ± 9

Continuous variables are presented as mean ± SD. BP: blood pressure; HDL: high density lipoprotein; LDL: low density lipoprotein. Statistical differences between groups were tested using Anova. Paired t-test was used for within-group comparison of visit 1 vs. 2, where *** p < 0.001; ** p < 0.01; * p < 0.05 (2-tailed).

### Changes in IMT with time

There was no statistically significant difference in left or right rIMT measured in healthy controls at visit 1 compared with visit 2 (Table 3). In the sub-set analysis, ACS subjects (n=117) had a significantly higher average rIMT (average of left and right rIMT) compared to healthy controls at visit 1 (0.36±0.07 vs. 0.30±0.04, p< 0.0001). There was no statistically significant difference in average rIMT at visit 2 for healthy controls compared with ACS subjects (0.29±0.05 vs. 0.30±0.07). There were no detectable differences between Common carotid IMT measured at visit 1 and 2 (0.07±0.02 and 0.07±0.01 respectively). Healthy controls had a significantly lower average cIMT at visit 1 and 2 compared with ACS subjects (0.07±0.02 vs. 0.80±0.21, p < 0.0001 and 0.07±0.01 vs. 0.81±0.25, p < 00001 respectively).

**Table 3:** Regression of Radial artery high-resolution ultrasound parameters in ACS subjects

	Parameter	Visit 1	Visit 2	Median change (IQR)	p-value
Healthy Control(n = 39)	Intima media thickness Right (mm)	0.31±0.05	0.30±0.05	−0.008 (−0.04 to 0.01)	ns
	Intima media thickness Left (mm)	0.29±0.05	0.28±0.05	−0.004(−0.06 to 0.02)	ns
ACS (sub-set) Subjects(n = 117)	Intima media thickness Right (mm)	0.35±0.08	0.29±0.08	−0.05(−0.32 to 0.17)	< 0.0001
	Intima media thickness Left (mm)	0.37±0.08	0.31±0.09	−0.06(−0.22 to 0.23)	< 0.0001

Data are expressed as mean ± SD or as median (IQR). Paired t-test was used for the comparison of visit 1 vs. 2.

**Figure 1:**
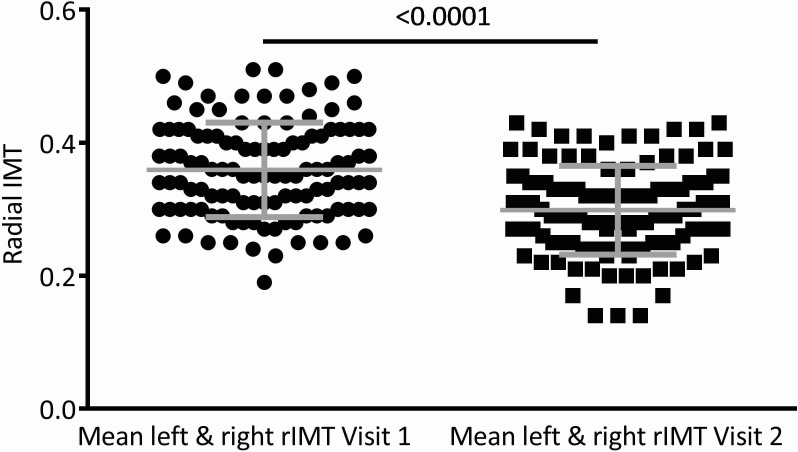
Radial artery intima media thickness (rIMT) measured on the right and left arm and averaged (mean left and right rIMT) at visit 1 and visit 2 At 4 months post ACS (visit 2), rIMT was decreased compared with rIMT at <1 month post ACS (visit 1).

In the subset analysis of 117 ACS subjects, radial IMT at visit 1 was significantly higher than rIMT at visit 2 (4 months post ACS), (p < 0.0001) (Figure 1). There were no detectable differences between Common carotid IMT measured at <1 month post ACS and at 4 months (0.80±0.21 vs. 0.81±0.25).

### Relationship between rIMT and CVD risk biomarkers

In the whole cohort (n= 256) who had rIMT investigated at visit 1, we first examined the relation between rIMT, clinical characteristics and blood biomarkers (NT-proBNP, and GDF-15) known to be associated with cardiovascular disease [16]. Furthermore, we examined the relation between rIMT and kidney biomarkers creatinine and cystatin C and inflammatory biomarkers CX3CL1, E-selectin and MPO. As shown in Table 4, rIMT significantly correlates with age, BMI, and gender. A number of biomarkers; NT-proBNP (cardiac), CX3CL1, MPO, GDF-15 and E- selectin (inflammatory), creatinine and cystatin C (kidney) and HDL-cholesterol significantly correlates with rIMT measured acutely post ACS (Table 4). We further performed a multivariate analysis adjusted for age, gender, BMI, HDL-cholesterol and diagnosis hypertension. After adjustment rIMT was significantly related to creatinine, Cystatin C, E-Selectin, CX3CL1, MPO and cIMT.

**Table 4:** Univariate and multivariate ^a^ correlation between conventional risk factors, patient demographics, biomarkers and average of right and left radial IMT measured within 1 month post ACS (visit 1)

Parameter	Univariate R	Univariate R^2^	Adjusted R^2^	n
Age	0.18**	0.03**	-	256
Gender	0.22**	0.05**	-	256
BMI	0.27***	0.07***	-	209
SBP	0.13	0.02	-	216
DBP	0.04	0.00	-	216
HDL-cholesterol	−0.22**	0.05**	-	213
LDL-cholesterol	0.02	0.00	-	213
Triglycerides	0.12	0.01	-	213
Cholesterol	−0.03	0.00	-	213
Creatinine	0.22**	0.05**	0.19*	213
Cystatin C	0.21**	0.05**	0.18*	216
Troponin T	0.09	0.01	-	144
NT-proBNP	0.17*	0.03*	-	216
GDF-15	0.21**	0.04**	-	214
E-selectin	0.21**	0.05**	0.18*	216
CX3CL1	−0.21**	0.05**	0.19*	214
MPO	−0.21**	0.04**	0.19*	214
Common Carotid IMT	0.28***	0.08***	0.19*	198

Univariate R: correlation coefficient, Adjusted R^2^: coefficient of determination after adjustment for age, gender, BMI, HDL and diagnosis of hypertension. *** p < 0.001; ** p < 0.01; * p < 0.05 (2-tailed). BMI: body mass index; SBP: systolic blood pressure; DBP: diastolic blood pressure; HDL: high density lipoprotein; LDL: low density lipoprotein; NT-proBNP: N-terminal pro b-type natriuretic peptide; GDF-15: growth differentiation factor 15; CX3CL1: C-X3-C motif chemokine ligand 1; MPO: myeloperoxidase; IMT: intima media thickness.

### Comparison of cardiac, kidney and inflammation biomarkers in healthy controls and ACS subjects

We analyzed cardiac biomarkers Troponin T and NT-proBNP at visit 1 and 2 for healthy controls and ACS subjects. Troponin T and NT-proBNP was significantly lower at visit 2 compared with visit 1 in ACS subjects, however there were no significant difference for healthy controls (Table 5). As expected, healthy controls had a significantly lower Troponin T and NT-proBNP compared with ACS subjects both at visit 1 and 2 (p< 0.0001 and p< 0.0001, respectively).

**Table 5:** Cardiac, kidney and inflammation biomarkers measured at visit 1 and repeated at visit 2 in sub-set ACS subjects and healthy controls

	Healthy control Visit 1	Healthy control Visit 2	ACS (sub-set) subjects Visit 1	ACS (sub-set) subjects Visit 2
Troponin T	4.64 ± 0.45 (n= 38)	4.65 ± 0.43 (n= 37)	29.6 ± 2.95 (n= 63)	10.50 ± 0.76*** (n= 105)
NT-proBNP	304 ± 33.23 (n= 38)	315 ± 45 (n= 37)	3941 ± 451 (n= 115)	2353 ± 324*** (n= 110)
Cystatin C	1356 ± 33 (n= 38	1375 ± 38 (n= 37)	1618 ± 38 (n= 115)	1597 ± 35 (n= 110)
Creatinine	82.29 ± 1.85 (n= 38)	81.59 ± 1.87 (n= 37)	89.27 ± 1.71 (n= 113)	88.72 ± 1.64 (n= 113)
GDF-15	7.78 ± 0.04 (n= 39)	7.92 ± 0.04) ** (n=37)	8.44 ± 0.06 (n= 113)	8.28 ± 0.05*** (n= 110)
E-selectin	13.64 ± 0.80 (n= 38)	13.87 ± 0.76 (n= 37)	13.93 ± 0.57 (n= 115)	14.49 ± 0.62 (n= 110)
CX3CL1	5.02 ± 0.28 (n= 39)	5.11 ± 0.32 (n=37)	4.89 ± 0.54 (n= 113)	4.56 ± 0.50*** (n= 110)
MPO	4.17±0.04 (n=39)	4.32±0.04** (n=37)	4.24±0.04 (n= 113)	4.01±0.03*** (n= 110)

Data are expressed as mean ± SEM. Paired t-test for parametric or Wilcoxon signed rank test for non-parametric test was used for the within-group comparison of visit 1 vs. 2 where *** p < 0.001; ** p < 0.01; * p < 0.05 (2-tailed).

There were no significant changes in cystatin C and creatinine at visit 2 compared with visit 1 for both ACS subjects and healthy controls. However, healthy controls had a significantly lower cystatin C and creatinine at visit 1 and 2 compared with ACS subjects (cystatin C p< 0.0001 and p< 0.0001, and creatinine p=0.0281 and p=0.0215, respectively).

In healthy controls, GDF-15 and MPO were significantly higher at visit 2 compared with visit 1, and in ACS subjects were significantly lower at visit 2 (Table 5). Healthy controls had a lower GDF-15 at visit 1 and visit 2 compared with ACS subjects (p< 0.0001 and p=0.001 respectively). At visit 1, there were no significant differences for MPO between both groups, however at visit 2 ACS subjects had a significantly lower MPO compared with healthy controls (4.01±0.03 vs. 4.32±0.04, p< 0.0001). There were no significant differences in E-selectin overtime for healthy controls and ACS subjects and there were no significant differences between both groups at either time point. In ACS subjects, CX3CL1 was significantly lower at visit 2 compared with visit 1, however there was no significant difference over time in healthy controls. There were no significant difference between healthy controls and ACS subjects at visit 1, however at visit 2 ACS subject had a significantly lower CX3CL1 compared with healthy controls (p< 0.0001) (Table 5).

### Biomarkers that are associated with increased or reduced rIMT

**Figure 2:**
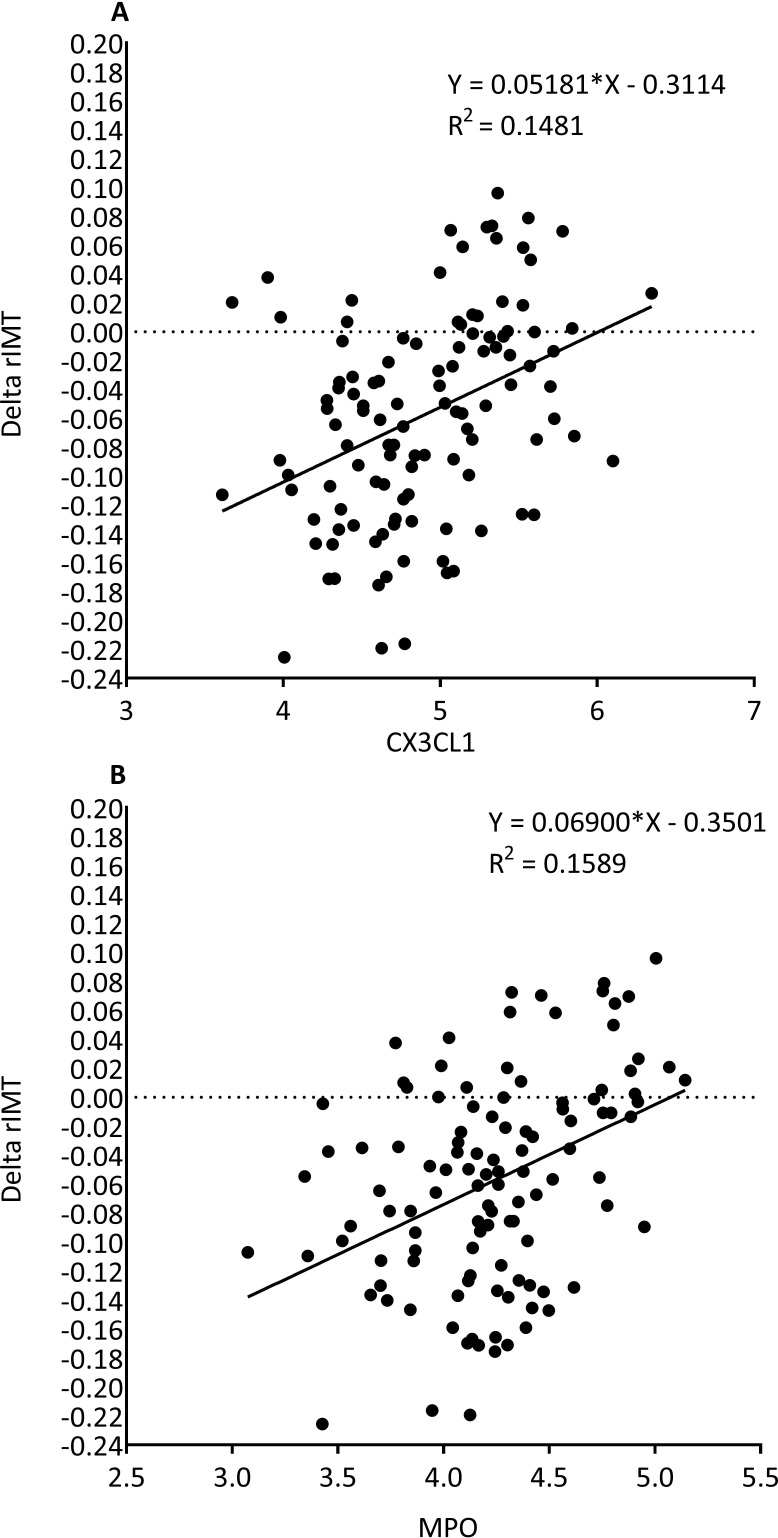
Linear regression graphs showing changes in radial IMT (increased or decreased rIMT over 4 months) is associated with CX3CL1 **(A)** and MPO **(B)** measured at less than 1 month post index event.

We further analyzed the relation between rIMT and inflammatory biomarkers known to play a role in atherosclerosis in the 117 repeatedly investigated ACS subjects. In the regression model CX3CL1 and MPO at visit 1 are associated with regressing or progressing rIMT r=0.38 (p< 0.0001) and r=0.41 (p< 0.0001) respectively. Figure 2 shows the linear regression graphs of CX3CL1 and MPO and subjects with regressing or progressing rIMT.

## DISCUSSION

In this study we have successfully measured rIMT in subjects after ACS and in healthy subjects. We show that rIMT regresses 4 months post ACS in both left and right radial artery, and is unchanged in healthy subjects within 4 months. As anticipated, we show that healthy controls have a lower rIMT and cIMT measured at baseline compared with ACS subjects, since we have previously shown that rIMT tracks with age and disease severity [10, 11, 13]. We also show that higher rIMT measured within 1 month of ACS correlates with elevated cardiovascular biomarkers creatinine, Cystatin C, E-selectin, CX3CL1 and MPO after adjustments for potential confounders. This suggests an interplay with inflammatory markers and progression of atherosclerosis. Furthermore, we show that inflammatory biomarkers CX3CL1 and MPO measured within 1 month of ACS are directly associated with rIMT. In healthy controls, we show rIMT and cIMT comparable to data previously published [13].

The intima-media complex is composed of the tunica intima and the tunica media. Intima changes may reflect hyperplasia or fibro-cellular hypertrophy in the intima [17], and early changes in the atherosclerosis process. Media thickening reflects hypertrophy and is accelerated by the presence of risk factors for atherosclerosis, importantly hypertension [17, 18]. Intima-media thickness is therefore a combination of the above mentioned processes and thus a good composite marker of cardiovascular risk. The endothelial glycocalyx (EG) is between flowing blood and the endothelial cell lining, and is suggested to be important for shielding the vascular wall from direct exposure to blood flow, contributes to vascular permeability barrier in capillaries, and exhibits anti-adhesive properties [19, 20]. In ACS, there is evidence suggestive of EG damage [21], and atherosclerosis per se has been linked to a reduction of the total EG volume, which was improved with short-term statin therapy [22]. Furthermore in ACS, it is know that there could be endothelial swelling which contributes to microvascular complications post PCI [23]. The composite of these above mentioned mechanisms will result in an acutely increased intima media thickness post PCI, therefore could explain our findings of higher rIMT measured acutely post index event.

Given that coronary artery disease continues to be a major health care burden [24], despite established therapies, sensitive tools are needed in clinical trials of newly developed therapies that will aid the selection of most promising therapies. In this study, we have established a relationship between rIMT and biomarkers (creatinine, cystatin C and MPO) which have been suggested to be indicative of poor prognosis in populations with acute coronary syndrome [25–27]. The relationship between increasing burden of atherosclerosis measured by intravascular ultrasound and adverse clinical outcomes have been shown [28]. Our results showing correlation between rIMT and cIMT supports the notion that atherosclerosis is a systemic disease and non-invasively determined rIMT is a potential tool reflecting this systemic nature, thus could be a surrogate marker for coronary artery atherosclerosis [11]. Unsurprisingly, we see correlations between rIMT and age, BMI and gender, which are known to contribute to atherosclerosis and these relationships with rIMT has previously been shown [10]. We see an inverse correlation between HDL-cholesterol and rIMT, but not with LDL-cholesterol. A plausible explanation could be that there could be confounding effects from recommended intensive statin treatment in this patient population, which will mainly have an effect by lowering LDL-cholesterol levels. Furthermore, high HDL-cholesterol is associated with low cardiovascular risk and is suggested to be protective against the development of atherosclerotic disease [29–31], hence the reverse association of HDL with rIMT is conceivable. The reverse association for CX3CL1 and MPO observed within 1 month post ACS remains highly speculative without good understanding currently. Although the lipid profile for healthy controls were higher than for ACS subjects, these values were comparable to published data for the age group studied [32].

We show that rIMT regresses at 4 months post ACS in a relatively small patient cohort. Although we do not have data on the use of statins at 4 months post ACS in this study, we hypothesize that the decreased rIMT 4 months after ACS may be a result of beneficial effect on the vasculature of secondary prevention medication (e.g. statins). In patients with atherosclerotic cardiovascular disease, the standard of care is the use of high-intensity statin therapy [33–35]. The standard of care post ACS in Sweden is also that all patients post PCI receive statin and 75mg aspirin. This is following the current European guidelines which recommends dual antiplatelet therapy of acetylsalicylic acid (Aspirin) plus a P2Y12 inhibitor (i.e., ticagrelor, prasugrel, ticlopidine or clopidogrel) to reduce the risk of thrombus formation in ACS patients [33, 35–37]. Furthermore, at the time of ACS, there is a heightened systemic inflammatory status [56], and in our study this is indicated by the associations of inflammatory markers (E-selectin, CX3CL1 and MPO) with rIMT after adjustments for clinical risk factors. Increased inflammation will trigger a vascular swelling response, which is likely reflected in the higher rIMT measured within 1 month post ACS. It is therefore expected that with the passage of time, intervention and treatment, this vascular swelling should improve. We also observed correlation between rIMT and creatinine, Cystatin C, which further verifies that rIMT is a surrogate marker for systemic disturbance. Creatinine and Cystatin C are both an index of renal function [38, 39], and Cystatin C has been shown to predict outcomes in patients with acute coronary syndrome [14, 40].

Associations between inflammation and increased cIMT has been shown [41–43]. We demonstrate here that there is a direct relationship between levels of inflammatory biomarkers CX3CL1 and MPO measured within 1 month of PCI and individuals who have increased or reduced radial IMT over time. This is an important association given that the progression of atherosclerosis is determined by the derangement of the equilibrium of immune responses [44]. On the endothelium CX3CL1 production induces leucocyte arrest, and may play a role in macrophage and T-cell recruitment to growing lesion, which furthers the progression of atherosclerotic lesion development and is also suggested to mediate pathological processes in cardiovascular disease [45–47]. MPO has been suggested to be a mediator of endothelial dysfunction and atherosclerosis, and elevated levels are associated with the presence of coronary artery disease, and predicts risk in ACS [48, 49]. Furthermore, it has been suggested that MPO binding to the EG decreases the electrostatic repulsion between the endothelium and leukocyte's surface, thereby inducing leukocyte recruitment, thus promoting inflammatory conditions [50]. Further research is required to enhance our understanding of how leucocyte driving biology drives radial artery atherosclerosis.

### Study strengths and limitations

To our knowledge, this is the first study that showed regression of rIMT measured using ultra-high-resolution ultrasound after the treatment of coronary stenosis. Coronary atheroma measured using invasive intravascular ultrasound has previously been shown to regress following high-intensity statin therapy [51, 52]. We believe that with the considerable improvement of the spatial resolution in the new ultrasound platform, rIMT could be an alternative or a complementary method to follow vascular structural changes over a relatively short time in much smaller patient populations.

A limitation is the lack of available data on the use of statins after PCI, and the low population size. The observed relationship between changes in rIMT and CX3CL1 and MPO are relatively weak possibly due to the explorative nature of these biomarkers and also the small population size. In the current study it is also difficult to dissect potential effect of specific medication on rIMT, therefore future randomized clinical trial is warranted to address this. Further, we included an apparently healthy control group, mainly to serve as a time control rather than to be compared with the patient population, due to the obvious difference between the two groups, in terms of age, gender distribution, medication etc.

In conclusion, in this study we used lipid profile, established cardiovascular biomarkers and explorative inflammation biomarkers to re-verify mechanistic correlates to rIMT. To further our understanding, we explored associations between rIMT and novel biomarkers CX3CL1 and MPO which are implicated in leucocyte mediated inflammatory responses. Taken together lipid profile, kidney and inflammatory markers CX3CL1 and MPO are associated with rIMT status, which supports our hypothesis that rIMT could serve as a surrogate marker for atherosclerosis. We present data showing that radial IMT is reduced after 4 months post ACS and secondary prevention medication. This suggests that rIMT could be used as a tool to monitor potential response to intensified medical therapy or as a new vascular surrogate marker for atherosclerosis in intervention studies.

## MATERIALS AND METHODS

### Study population

256 subjects with significant coronary atheroma and underwent PCI including stent due to acute coronary syndrome at the Department of Cardiology, Sahlgrenska University Hospital, Gothenburg, Sweden were invited to participate in the study, performed during 2010 to 2014. To correct for potential time-dependent changes in all the parameters assessed, we also recruited 39 elderly healthy controls, who underwent a similar study protocol. To be enrolled as healthy controls, the subjects were screened to be free from previous or current diseases requiring medication, dyslipidemia, hypertension, diabetes, and pathological exercise ECG test. Written informed consent was obtained before entry into the study and participants were examined within 1 month of presenting with ACS (visit 1), or baseline for healthy controls (visit 1). All 256 subjects had rIMT and cIMT measured within a month of undergoing PCI, and of the 256 subjects a sub-set of 117 subjects had rIMT repeated at 4 months after ACS or 4 months after baseline visit for healthy controls (visit 2). Methodology and feasibility of rIMT has previously been shown [10]. Demographic data, including history of diabetes, hypertension, hyperlipidemia, previous coronary artery bypass grafting, previous *PCI*, smoking status and current cardiovascular medication at the time of ACS were collected from the patient medical records. The study was approved by the local ethics committee at the University of Gothenburg and complies with the declaration of Helsinki.

The exclusion criteria included serious illness other than cardiovascular disease, and inability to give informed consent.

### High-resolution ultrasound of the radial artery

Both left and right radial arteries were examined non-invasively in longitudinal view, with near and far walls clearly visible, using a 50 MHz transducer with a resolution down to 30 μm (Vevo 2100 VisualSonics, Inc, Toronto, Ontario, Canada). To standardize rIMT image acquisition, previously published protocol was used [13]. Briefly, the transducer was placed in the antebrachial anterior region at the second skinfold, 1-2 cm proximal to the Palma manus. Digital cine-loops of three consecutive cardiac cycles in B-mode were stored for offline analysis. IMT is defined as the distance between leading edges of the lumen-intima and media-adventitia interfaces [53]. IMT measurements were standardized and performed in peak systole; defined as the frame in systole where the artery had its largest diameter in a cine loop [10]. All offline measurements were done by a single operator who was blinded to the clinical characteristics of the subjects.

### Carotid artery intima media thickness

Left and right carotid arteries were examined in the longitudinal view, with near and far walls visible, using a 8 MHz linear transducer (Siemens, Acuson Sequoia 512, Mountain View, California), conforming with recommendations [53]. Measurements of common cIMT was performed 1 cm proximal to the bifurcation, in peak systole and in two different heart beats. Digital cine-loops of three consecutive cardiac cycles were stored for offline analysis. Mean values of the left and right cIMT were used in statistical analysis.

### Laboratory analysis

Overnight fasted blood samples were drawn within 30 minutes of rIMT measurement. All biochemical analyses were performed at the Department of Clinical Chemistry, Sahlgrenska University Hospital, Gothenburg or AstraZeneca R&D, Gothenburg, using standard commercially available kits, according to the manufacturer's protocols. To evaluate lipid profile, we measured serum triglycerides, cholesterol, high density lipoprotein (HDL), low density lipoprotein (LDL) using standard photometric methods in all subjects. In addition to lipid biomarkers, known kidney biomarker Cystatin C was analyzed for kidney status using standard electro-chemiluminescence method, and creatinine analyzed using spectrophotometry. To investigate cardiovascular status, among known biomarkers; Troponin T and N-terminal of the prohormone brain natriuretic peptide (NT-proBNP), were analyzed using standard electro-chemiluminescence methods. Furthermore, growth differentiation factor-15 (GDF-15), Troponin T and NT-proBNP are well established risk biomarkers in the population studied [54]. Growth differentiation factor-15 (GDF-15), E-selectin, Fractalkine (CX3CL1) and myeloperoxidase (MPO) were analyzed using the using the Olink Bioscience (Uppsala, Sweden) Proseek multiplex CVD III panel (multiplexed proximity extension assay) according to the manufacturer's instructions [55]. All Proseek data are presented as arbitrary units (AU) in log^2^ values. E-selectin, CX3CL1 and MPO are inflammatory biomarkers, and were analyzed to investigate inflammatory status and associations with rIMT.

### Statistical analysis

Deviations in sample size for the various statistical analyses were due to differences in the availability of clinical demographic data, as well as missing values in some analyzed biomarkers/parameters. Analyses were performed using SPSS, (version 22.0, Chicago Inc., USA) and TIBCO Spotfire, (version 5, TIBCO Software Inc., Boston USA). A p-value of <0.05 (2-tailed) was considered significant. Values are displayed as mean ± SD for continuous variables and percentages for categorical variables. Out of the 256 subjects, a sub-set (n= 117) were completely investigated at visit 1 and visit 2 in regards to rIMT and cIMT and further analyzed in sub-analyses. The test of skewness was used to assess normal distribution. Non-normally distributed variables are presented with their median and interquartile range. Differences among continuous variables were analyzed using paired t test or Wilcoxon Signed rank test, as appropriate and statistical significance for categorical variables was calculated using the chi-squared test. Logistic regression (categorical data) or partial correlation was used in multivariate analysis adjusted for age, BMI, gender, HDL-cholesterol and diagnosis hypertension, selected based on statistical significance and know clinical relevance for the independent parameters.

## Abbreviations

ACS: acute coronary syndrome
BMI: body mass index
cIMT: carotid artery intima-media thickness
CVD: cardiovascular disease
GDF-15: growth differentiation factor-15
HDL: high density lipoprotein
LDL: low density lipoprotein
MPO: myeloperoxidase
NT-proBNP: N-terminal of the prohormone brain natriuretic peptide
PCI: percutaneous coronary intervention
rIMT: radial artery intima-media thickness
